# Recent advances in removal of Congo Red dye by adsorption using an industrial waste

**DOI:** 10.1038/s41598-022-10093-3

**Published:** 2022-04-12

**Authors:** Maria Harja, Gabriela Buema, Daniel Bucur

**Affiliations:** 1 Faculty of Chemical Engineering and Environmental Protection, “Gheorghe Asachi” Technical University of Iasi, 73 Prof.dr.doc. Dimitrie Mangeron Street, 700050 Iasi, Romania; 2 National Institute of Research and Development for Technical Physics, 47 Mangeron Boulevard, 700050 Iasi, Romania; 3 Department of Pedotechnics, Faculty of Agriculture, University of Life Sciences, 3, Mihail Sadoveanu Alley, 700490 Iasi, Romania

**Keywords:** Environmental sciences, Engineering, Materials science

## Abstract

The Congo Red dye was removed from a simulated textile wastewater solution using fly ash from a local power plant. The characterisation of fly ash was studied in detail by SEM, EDX, XRD, FTIR, BET surface area and TGA techniques. The influence of four parameters (contact time, initial concentration, adsorbent dose, and temperature) was analysed, the results showing that the adsorption capacity depends on these parameters. Thermodynamic and regeneration investigations as well are presented. The fit to pseudo-second-order kinetics models suggests that the removal process is a chemical adsorption. The Langmuir model fitted the experimental data, with a maximum adsorption capacity of 22.12 mg/g. The research is a preliminary case study that highlights that fly ash posed a very good potential as a material for Congo Red dye removal.

## Introduction

Water polluted with different contaminants, such as toxic heavy metals and dyes, has negative effects on human health^1^. The azo dyes (Congo red), a main class of synthetic colourants, are usually used in the textile manufacturing due to their properties, such as a large variety of shades, resistance to decolouring, and a lower energy consumption^2^. The azo dyes can have one or more chromophoric groups and aromatic rings. The π-conjugated azo bond characteristics and resonance make azo dyes very stable to light and aggressive environments; thus, they are recommended for chemical industries, textile dyeing, paper, cosmetics, and pharmaceutical, for example^3–7^. Annually, many types of dyes are produced worldwide in a quantity of up to 108 tons; this is especially true for azo dyes (60–70% of the total). The extensively usage of azo dyes leads to a large volume of wastewater that contains azo dye pollutants. Congo Red (diazo dye) is recognized as a carcinogen because it contains an aromatic amine in its structure. The presence of aromatic structures makes azo dyes resistant to natural degradation. Dyes remain in the environment for a long period and have negative effects on the fauna and flora^8–12^. Consequently, the treatment of water contaminated with Congo Red dyes is necessary.

Different methods have been proposed to remove Congo Red from polluted water, such as adsorption^1,13^, coagulation–flocculation^14^, ultrasound irradiation^15^, ion exchange^16,17^, mineralisation^2^ and photocatalysis^18^, etc. Of these, adsorption has a long history and will continue to be of great interest due to its advantages of low cost, large quantities of available adsorbents, high adsorption capacity, easy regeneration potential and minimum energy requirement^19–27^. The type and properties of adsorbent influence the adsorption process. More research has been realised to study the use of various adsorbents for the elimination of Congo Red (CR) dye from wastewater^28–33^.

Fly ash (FA), an alternative adsorbent for the waters contaminated with CR dye, is a waste material available in numerous countries at great amounts. The main constituents of FA include inorganic oxides such as SiO_2_, Al_2_O_3_ and Fe_2_O_3_ and small quantities of Na_2_O, MgO and K_2_O^34^.

**Figure 1:**
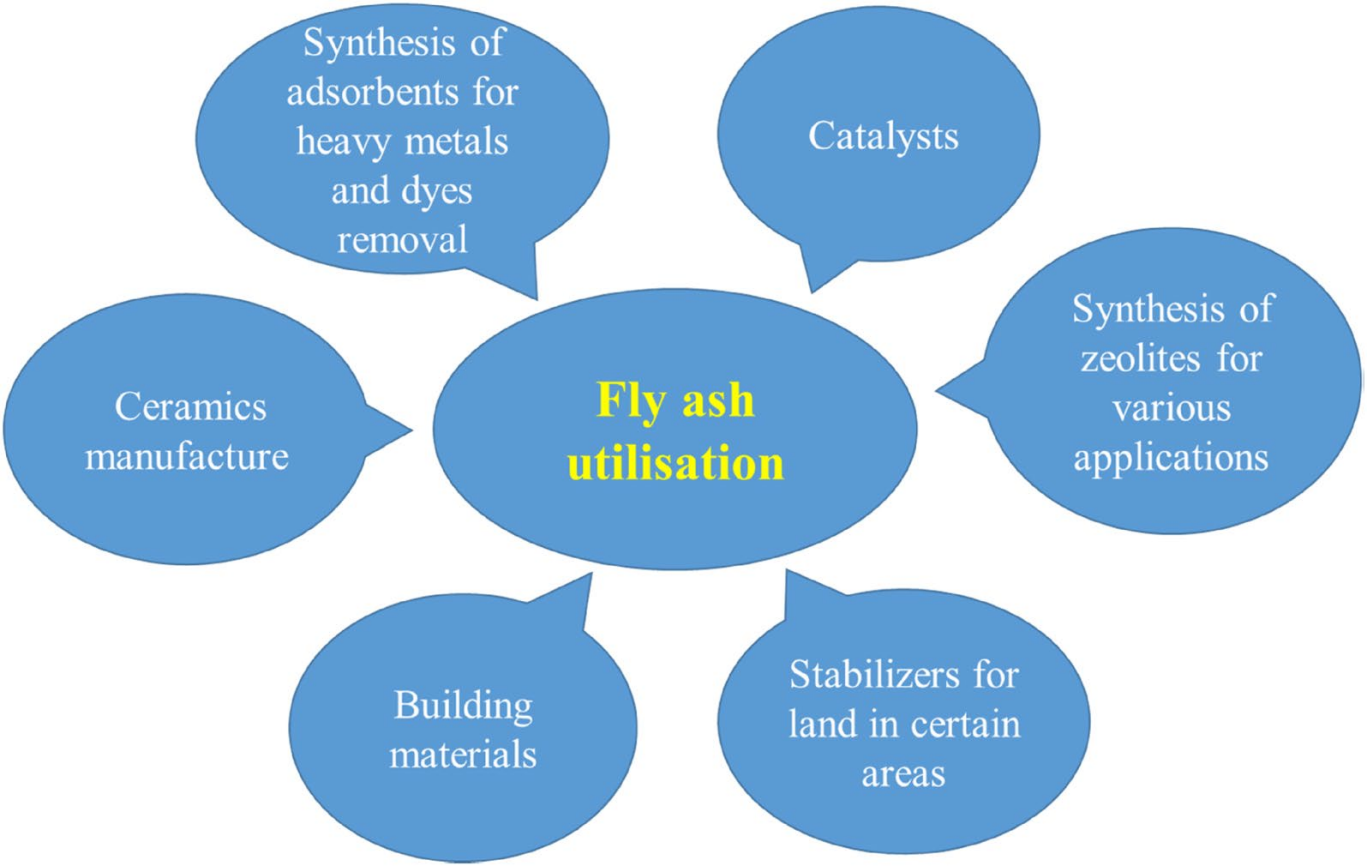
Fly ash utilisation.

Figure 1 shows the principal fields dedicated to the use of FA.

Of all possible uses, contaminant removal and the synthesis of adsorbents for contaminant removal represent interesting challenges. The ability of FA to remove pollutants has been demonstrated in several previous reports^35–39^.

This study has as objectives:

- A detailed characterisation of FA;
- Investigation of batch adsorption to analyse the influence of FA dose, contact time and initial CR dye concentration;
- Kinetic evaluation;
- Equilibrium evaluation.

In the current study an eco-friendly method is presented to demonstrate that an abundant industrial waste can be successfully used for elimination of CR dye from polluted water. The adsorption capacity of FA for Congo Red^40^ dye removal from simulated textile wastewater was investigated. However, FA and the modified ash adsorbents are not novel, but this study is the first to demonstrate the non-necessity of expensive modification in order to obtain adsorbents as effective as unmodified ash. The novelty of this paper is focused on the efficiency of the unmodified ash as efficient adsorbent for dye removal. The influences of contact time, FA dose and initial concentration over CR dye removal, adsorption isotherms and kinetics were analysed in a batch contact system. In order for the proposed method to be of lowest cost possible, the experiments were performed at ambient temperature and natural pH. The novelty and originality of the present work is based on the following criteria: (1) the use of Fly ash material as adsorbent for Congo Red dye without any modification; (2) the adsorption capacity data of unmodified FA as adsorbent presented in the specialized literature are lower; (3) to best of our knowledge there are no data regarding the capacity of a Romanian fly ash to remove Congo Red dye; (4) through this research information regarding the removal of Congo Red dye will be added to the existing literature.

## Results and discussion

### Adsorbent characterisation

#### SEM results

**Figure 2:**
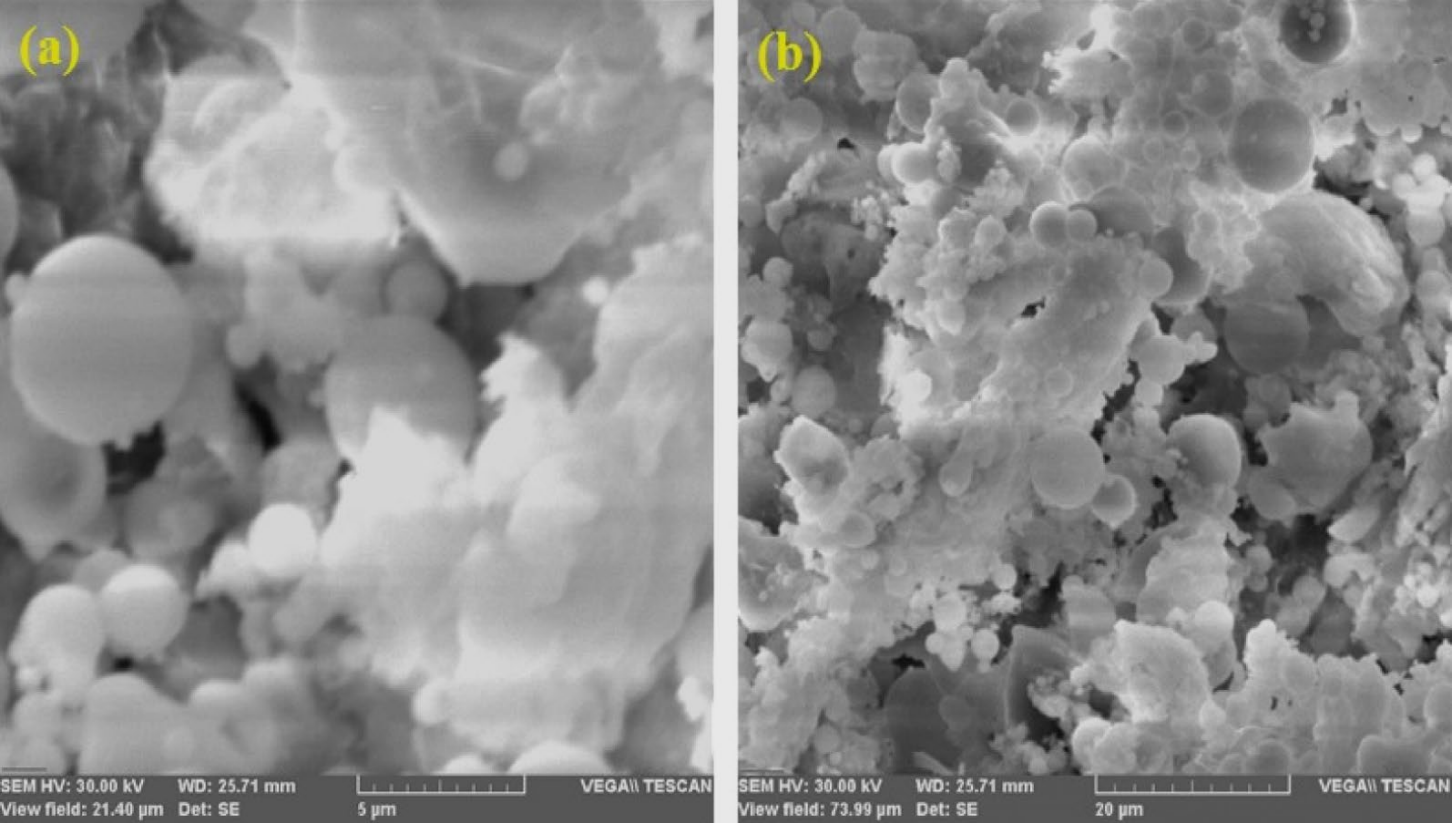
SEM images of fly ash.

Fly ash is a powder, light grey in colour. The morphology of FA is determined by the origin of the coal, combustion temperature and cooling rate. In this study the particle size was 1–200 μm. SEM images of FA are shown in Fig. 2. The particles consisted of spheres, hollow ceno-spheres and irregularly shaped particles. The images at two different resolutions show that the spherical particles are covered by a non-crystalline phase, formed in the combustion process and consisting of a vitreous phase^41^. Relatively small agglomerated particles, formed of an irregular amorphous phase, appeared due to particle contact or as a result of rapid cooling.

#### EDX results

**Figure 3:**
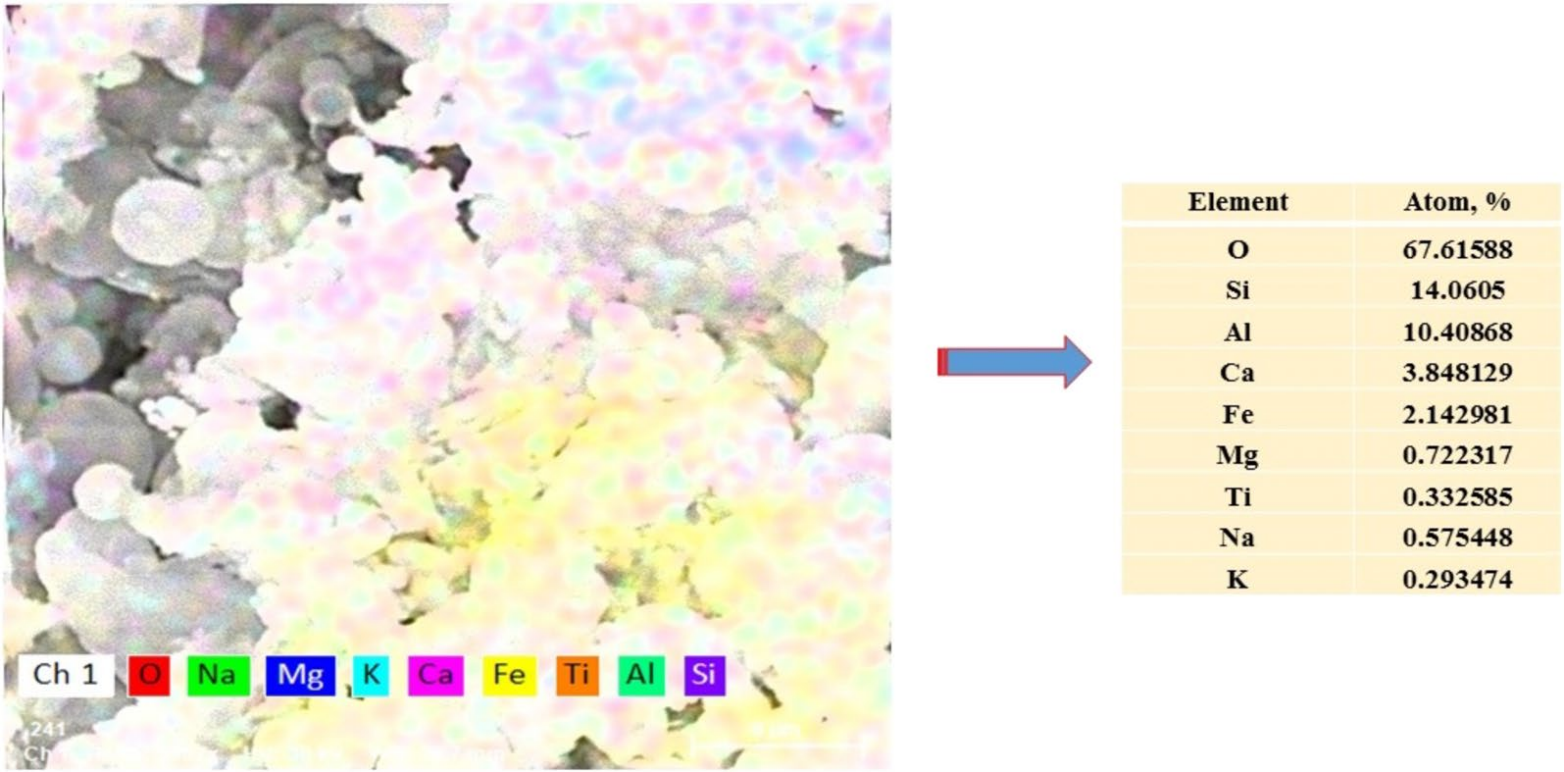
EDX analysis of fly ash.

Figure 3 shows the mapping diagram of the FA. Also, the content of each element is presented.

The element distributions (Si, Al, Mg, Ca and Na) of FA demonstrated that it is a low-calcium ash, composed mainly of silicate, Al-silicate and Fe-silicate components^42,43^.

#### FTIR results

**Figure 4:**
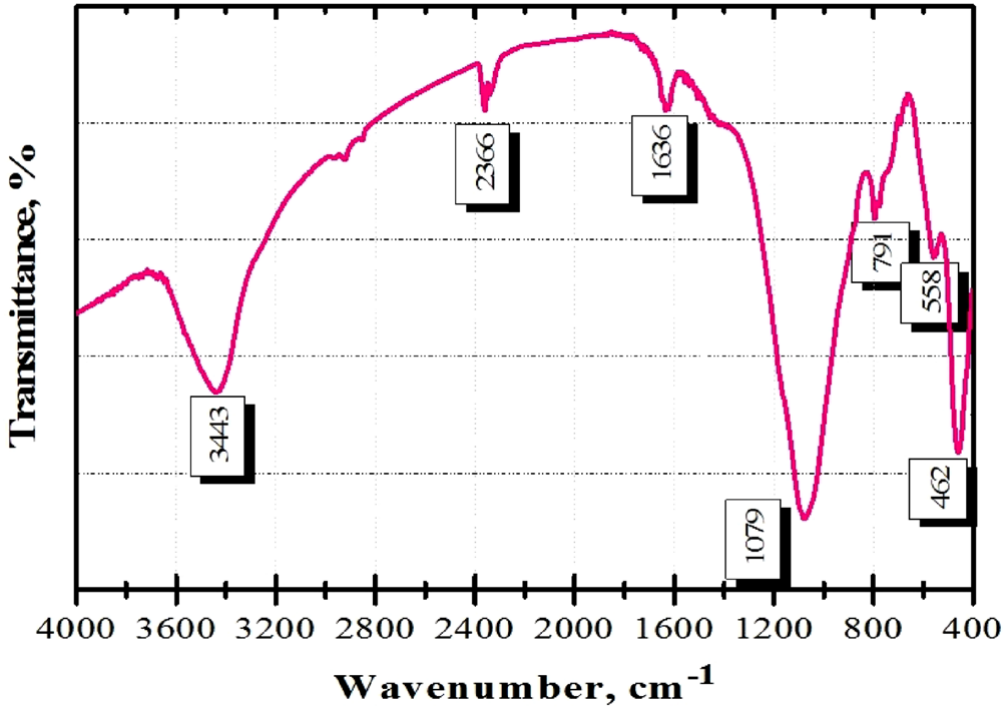
FTIR spectrum of fly ash.

The FT-IR spectrum of the adsorbent material is depicted in Fig. 4.

The functional groups of FA are as follows (Table 1):

**Table 1:** Functional groups assigned to wavenumbers.

Wavenumber, cm^−1^	Assignment
3443	Hydroxyl O–H stretch
2366	Al–O bond vibration
1636	O–H vibration
1079	Si–O vibration
791	Si–O–Si symmetric mode
558	Si–O–Al
462	Si–O–Si

From Fig. 4 and Table 1 it can be observe that FA shows one peak at ~ 3600 to 3000 cm^−1^, due to hydroxyl group; small quantities of water are also observed from the absorption at 1636 cm^−1^^44^. In the literature it is reported that the peaks from 1250 to 700 cm^−1^ present a characteristic region of the silica network^45^. In this study, the Si–O–Si symmetric band is at ~ 791 cm^−1^, and bands corresponding aluminates and silico-aluminates were also found, all in accordance with the EDX analysis (Fig. 3).

#### XRD results

**Figure 5:**
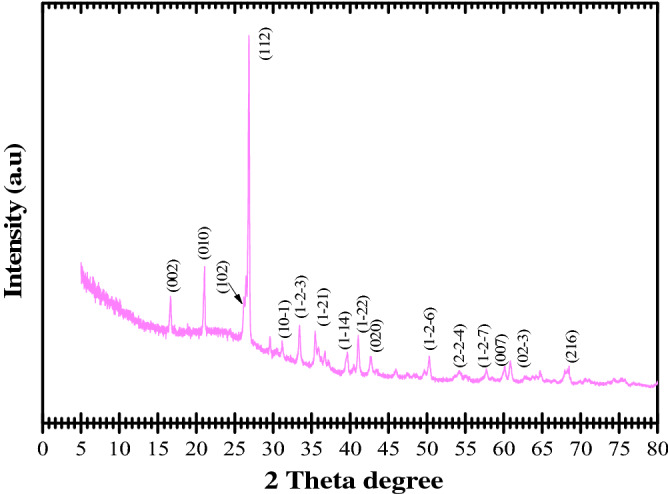
X-ray diffraction of fly ash.

The mineralogical composition was established by XRD analysis (Fig. 5).

According to X-ray data, two phases were found in the FA.

The first peak, at ~ 16.61° (002 plane), is characteristic of mullite. Also, the mullite phase is indicated by the peaks at ~ 26.19°, 31.12°, 33.51°, 35.52°, 41.07°, 54.29° and 60.07° which correspond to the (102), (10-1), (1-2-3), (1-21), (1-22), (2-2-4) and (007) planes, respectively.

The well-defined peaks at ~ 21.06°, 26.86°, 50.31°, 57.7°, 60.83° and 68.32° correspond to quartz and can be indexed to the (010), (112), (1-2-6), (02-3), (1-2-7), (02-3) and (216) reflections, respectively. The peaks at 39.55° → (1-14) plane and 42.69°→ (020) plane can also be attributed to quartz. The literature reports similar data^46^.

#### BET surface area results

**Figure 6:**
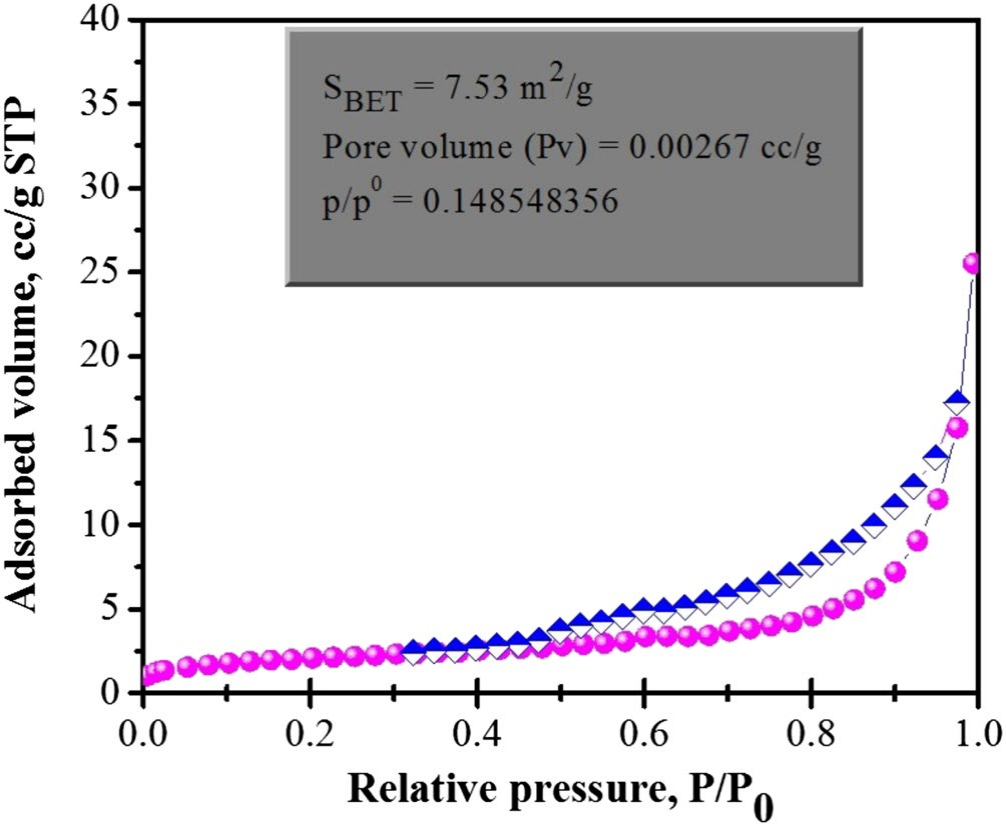
Nitrogen adsorption (magenta circles) and desorption (blue diamonds) isotherms of fly ash.

The BET surface area is a key factor affecting adsorption performance. The experimental results are revealed in Fig. 6. According to the data obtained the adsorption curve of FA corresponds to a Type IV isotherm, as per IUPAC classification.

The average value of pore size was 1.546 nm. The FA is a mesoporous material (average pore size 2–50 nm) based on the experimental data.

#### TGA results

**Figure 7:**
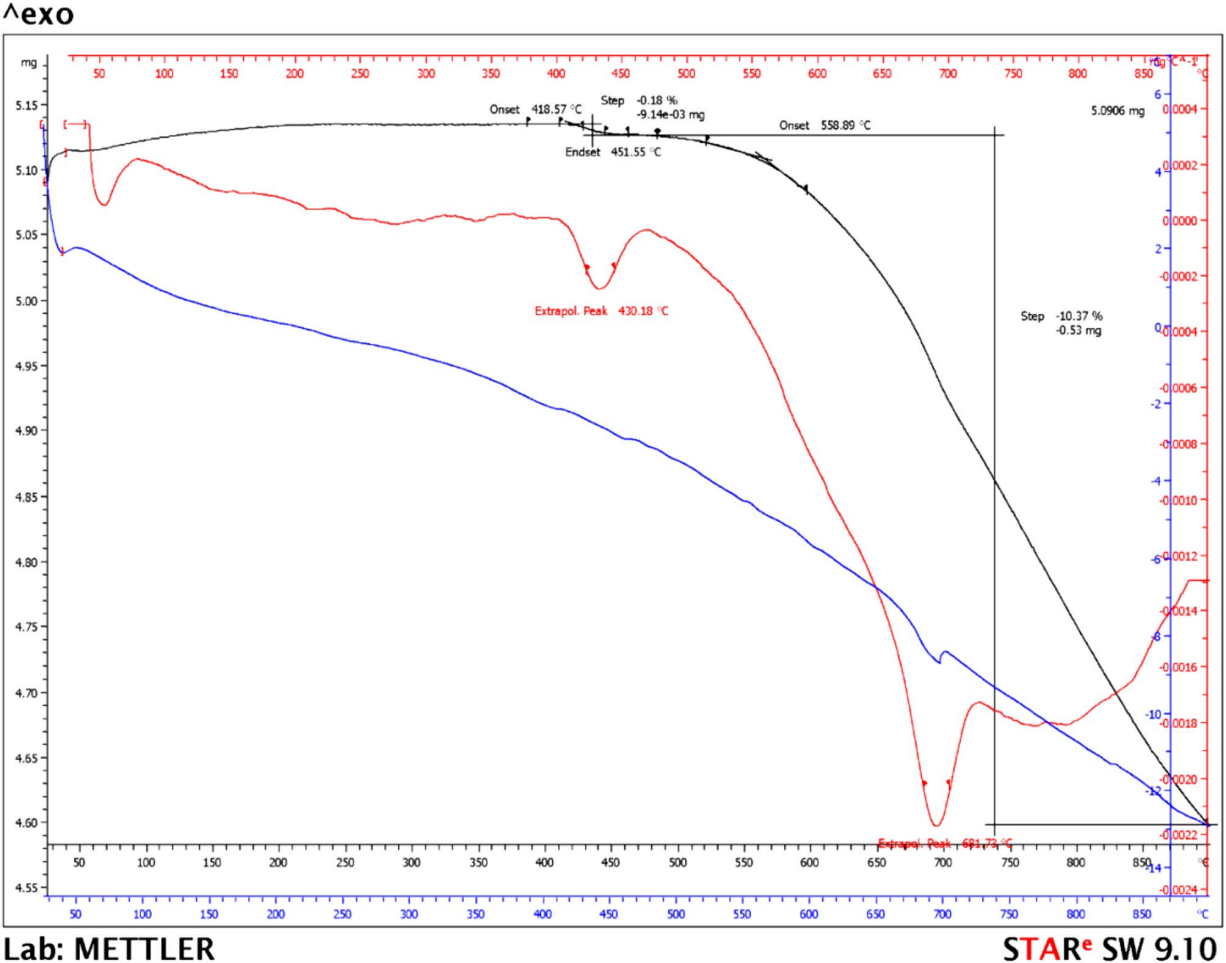
TGA of fly ash in N_2_ atmosphere (10 °C/min).

The results can be observed in Fig. 7.

The TGA results (Fig. 7) indicated that the FA showed a continuous mass loss. In the first stage a low mass loss (0.18%) was observed due to the physically bound water. Above 450 °C, the second step can be attributed to the release of chemically bound water. An important endothermic peak appeared at 681 °C due to decarbonation of calcium and magnesium carbonate. The CaO and MgO formed during the coal combustion process react with water, forming hydroxides, which then react with carbon dioxide in air and can determined the carbonation process. The temperature of the endothermic peak and the mass loss of confirmed this hypothesis. The FA used in this study had a total mass loss of 10.55%, and the results are in accord with the EDX and FTIR analyses and data reported in the literature^47,48^.

### A preliminary case study for CR dye adsorption by FA adsorbent

The adsorption behaviour was examined by varying the parameters (initial dye concentration, adsorbent dose and contact time). A relationship, with R^2^ = 0.9997, between absorbance and the CR dye concentration, was found at 498 nm (initial concentration 5–30 mg/L). The detection limit of CR dye was 5 mg/L.

The equilibrium adsorption capacity ‘*q*_*e*_’ was determined by Eq. (1), while the adsorption capacity was determined by Eq. (2):


#### Effect of FA dose

**Figure 8:**
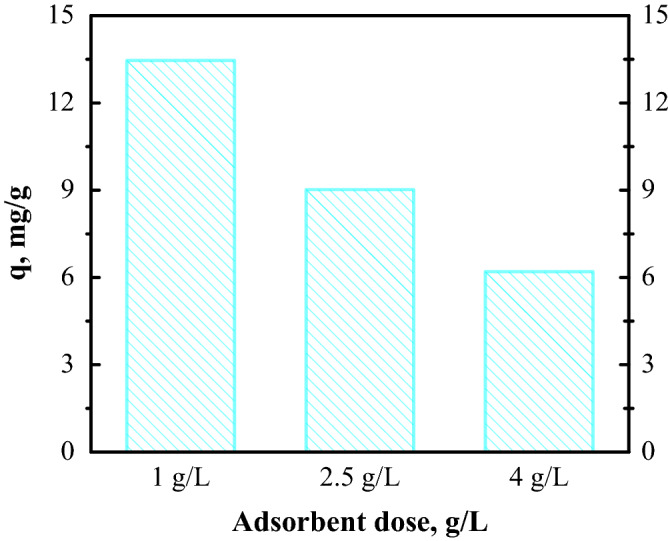
Congo Red dye adsorption vs. fly ash dose.

The study was carried out at three values of adsorbent dose (Fig. 8).

From the results it is evident that the *q*_e_ decreased (from 13.46  to 6.2 mg/g) with increasing adsorbent dose. An FA dose of 1 g/L was selected for further investigation. Analysing experimental data it was observed that at 4 g/L the removal percentage, R(%)^28^, was 82%, while after 6 g/L the R(%) was ~ 100%.

#### Effect of contact time

Contact time has a main influence in the adsorption process: a good contact time makes the adsorbent feasible in the treatment of contaminated water.

**Figure 9:**
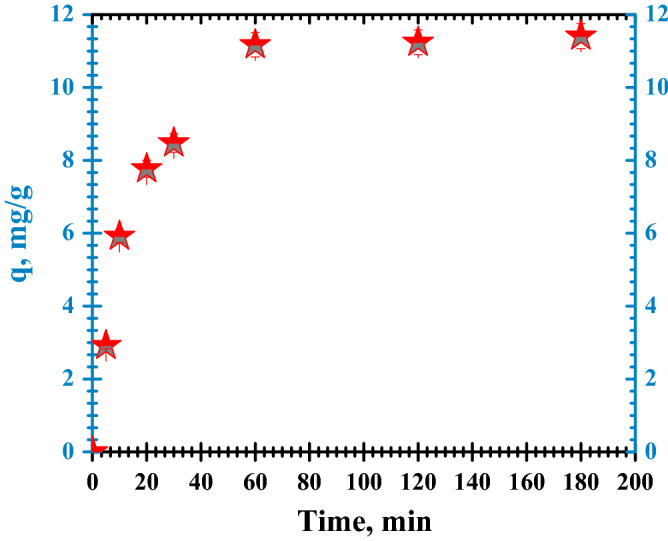
Congo Red dye adsorption vs. contact time.

This parameter was varied from 5 to 180 min. The results regarding the capacity of FA to remove CR dye are presented in Fig. 9.

As shown in Fig. 9, for the investigated adsorbent the adsorption capacity increased quickly in the first 30 min of contact time under the proposed operating conditions. The initial adsorption stage is rapid due to the number of available sites. Equilibrium was touched after 60 min (q = 11.4 mg/g).

#### Adsorption kinetics

**Figure 10:**
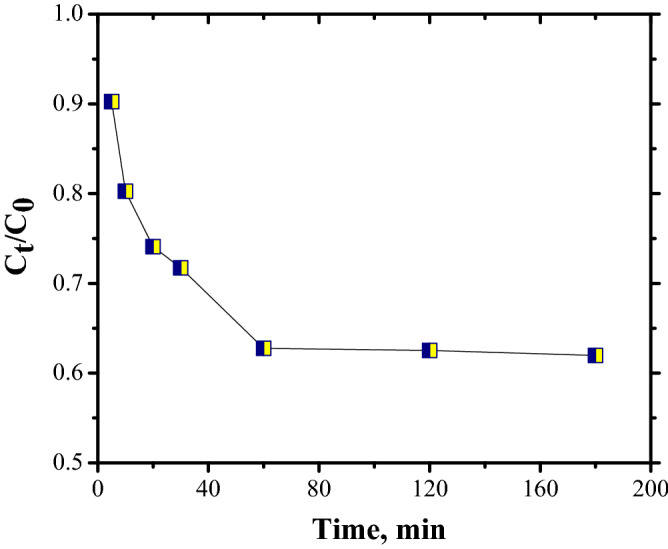
Kinetics of adsorption of Congo Red dye.

Figure 10 shows the variations in CR dye concentration in the liquid phase (C_t_/C_o_) in relation to time in contact with the FA adsorbent.

The experimental data were tailored to the pseudo-first-order (PFO), pseudo-second-order (PSO), and intraparticle diffusion (IP) models, Eqs. (3–5):

The preliminary adsorption rate (h) at t → 0 was determined according to Eq. (6):

6 $$h={k}_{2}{{q}_{e}}_{calc}^{2}$$

**Figure 11:**
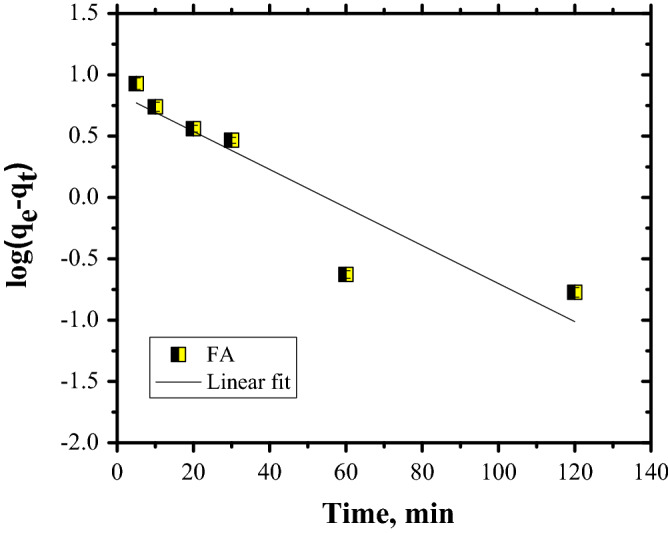
PFO kinetics of Congo Red dye adsorption.

**Figure 12:**
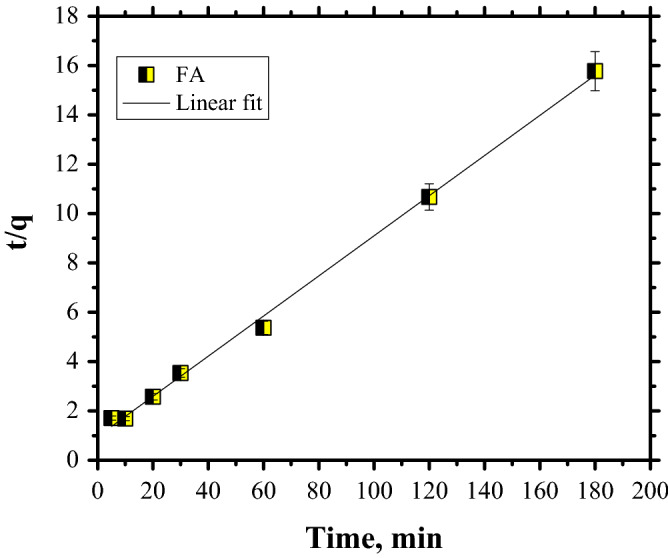
PSO kinetics of Congo Red dye adsorption.

**Figure 13:**
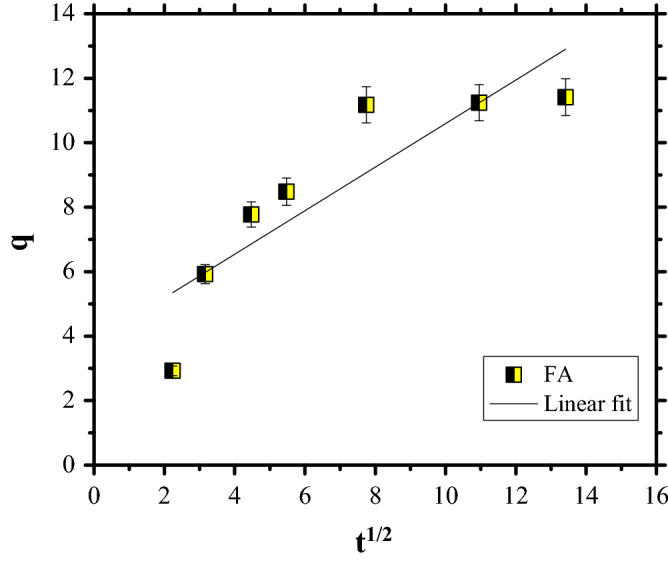
Intraparticle diffusion model of Congo Red dye adsorption.

The plots for CR dye adsorption are shown in Figs. 11, 12 and 13, while the kinetic parameters are in Table 2.

**Table 2:** Kinetics parameters for CR dye adsorption on FA.

q_e_, (mg/g)	PFO		PSO				IP diffusion		
	k_1_, (1/min)	R^2^	q_cal_, (mg/g)	k_2_ (g/mg min)	R^2^	h (mg/(g min)	k_i_, mg/g·min^0^^.5^	c	R^2^
11.41	0.0357	0.8534	12.3	0.0068	0.9977	1.03	0.6757	3.8375	0.7677

**Figure 14:**
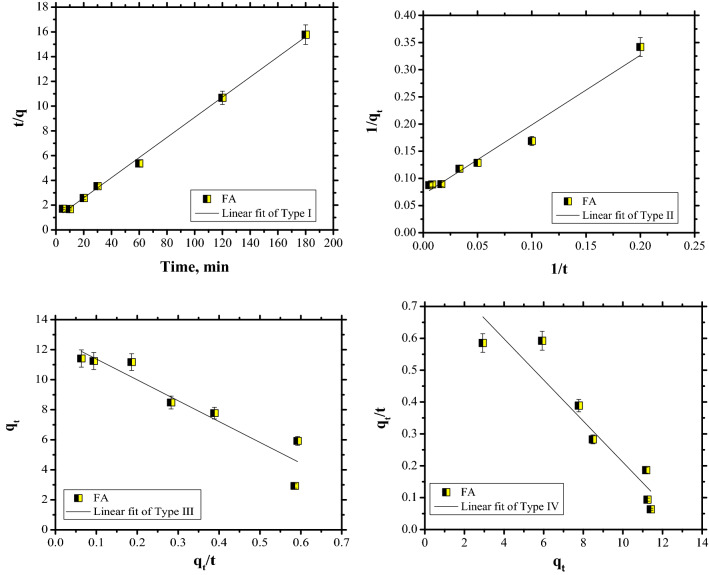
Pseudo-second-order model, Types I–IV.

Taking into account that the data are fitted by PSO kinetics it can be stated that the rate-limiting step of CR dye adsorption is a chemical adsorption process. Furthermore, the PSO kinetics were linearised in four linear forms (Fig. 14). The values of parameters for the four linearised versions, such as: q_e_ and k and the correlation coefficient, R^2^, are listed in Table 3.

**Table 3:** Linear form of PSO models.

	q_e_	k	R^2^
PSO constants → Type I	12.3	0.0068	0.9977
PSO constants → Type II	14.2	0.0039	0.9732
PSO constants → Type III	12.76	1.0872	0.8956
PSO constants → Type IV	13.27	0.0048	0.8956

The parameters **q**_**e**_ and **k** were determined from the intercept and slope of a straight line using the equations corresponding to each type^49^. By analysing the results presented in Table 4 was demonstrated that the adsorption kinetics of CR dye by FA are Type II PSO kinetics; Types III and IV are not indicated. The linearised version of Type I PSO kinetics represents the data since the correlation coefficient is closer to unity. The adsorption capacities using all four version of PSO model show a good fit with the experimental data.

**Table 4:** Parameters for Langmuir and Freundlich models.

Langmuir			Freundlich		
q_max_ (mg/g)	K_L_ (L/mg)	R^2^	K_F_ ((mg/g)/(L/mg)	1/n	R^2^
22.12	0.1366	0.9105	1.33	0.54	0.8941

#### Initial CR concentration effect

Four concentrations, between 10 and 50 mg/L, were analysed while maintaining the other parameters constant.

**Figure 15:**
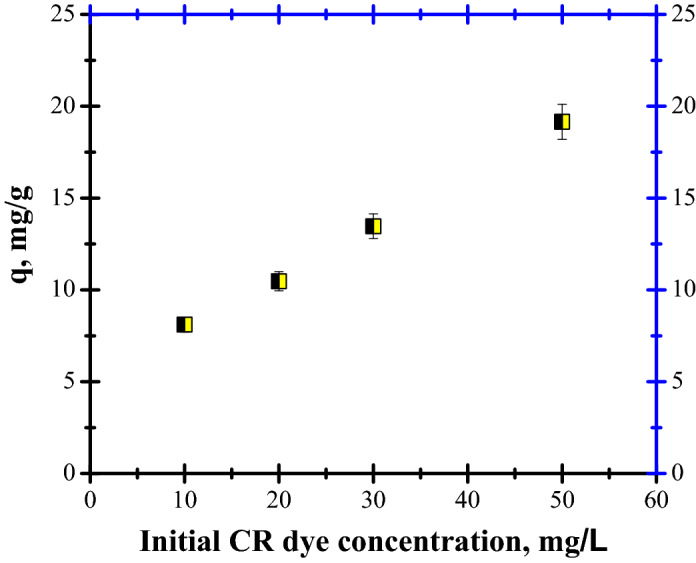
CR dye adsorption as a function of initial concentration.

The results regarding the capacity of FA to remove CR dye are presented in Fig. 15.

The results obtained from Fig. 15 illustrated that, under our experimental conditions, adsorption capacity increased from 8.1 to 19.2 mg/g with an increase in CR concentration from 10 to 50 mg/L. The concentration dependence of CR dye adsorption can be ascribed to a significant decrease in mass gradient between the FA and the solution under high CR concentration.

**Figure 16:**
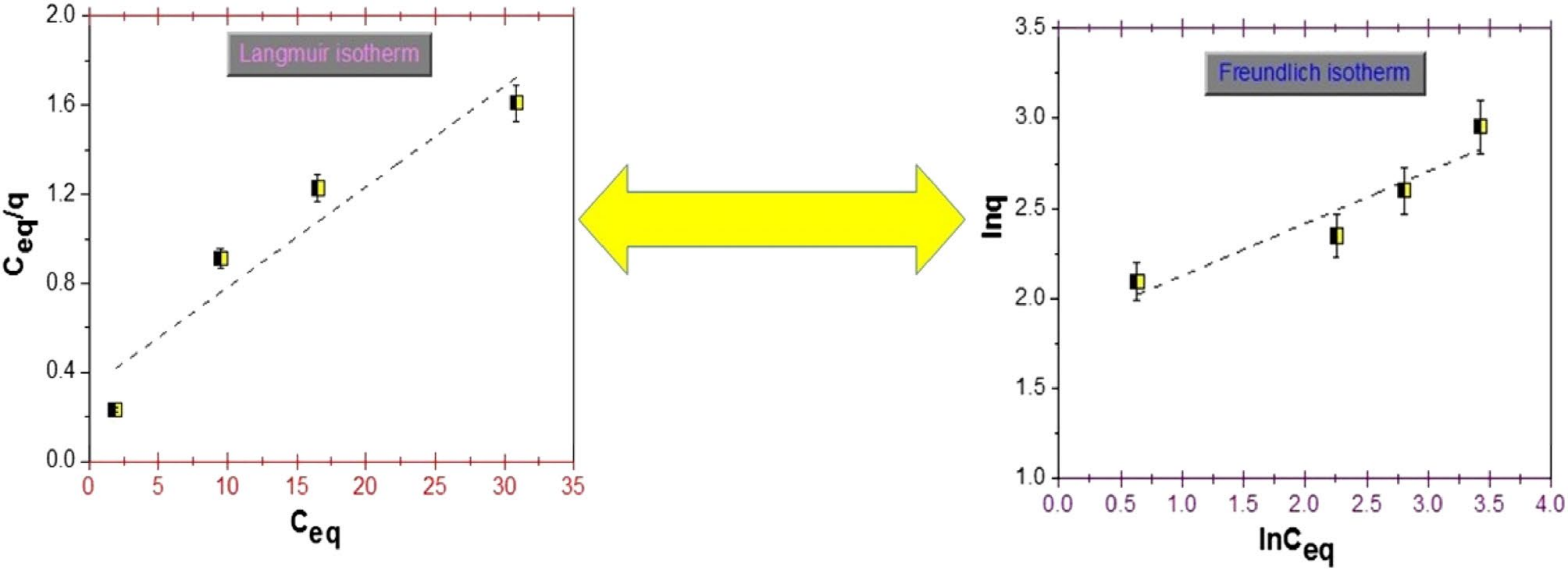
Langmuir isotherm (left) and Freundlich isotherm (right) for Congo Red dye adsorption by fly ash.

The type of interaction between the solute and FA adsorbent was established using two isotherms; the fits are presented in Fig. 16.

Table 4 presents the parameters values of the isotherms.

The data from the table above show that the Langmuir model fitted the results obtained.

#### Effect of temperature and adsorption thermodynamics

**Figure 17:**
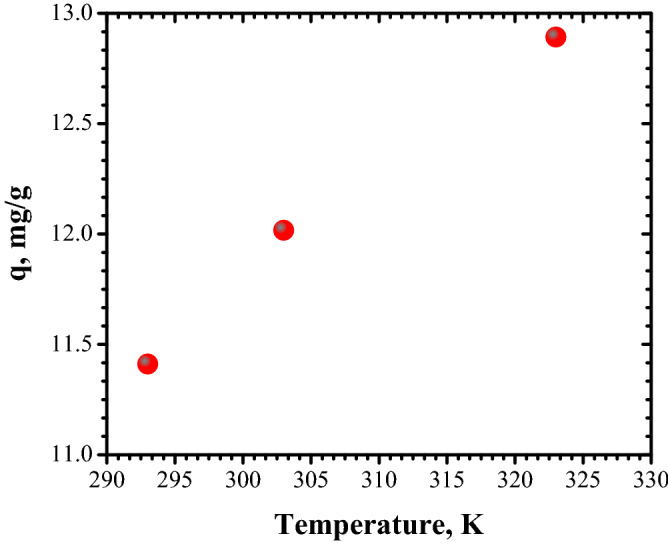
Effect of temperature on adsorption of Congo Red dye.

The effect of temperature on adsorption of 30 mg/L initial concentration of Congo Red dye on 1 g/L FA adsorbent dose was investigated. The experiments were completed at 293 K, 303 K, and 323 K (Fig. 17).

**Figure 18:**
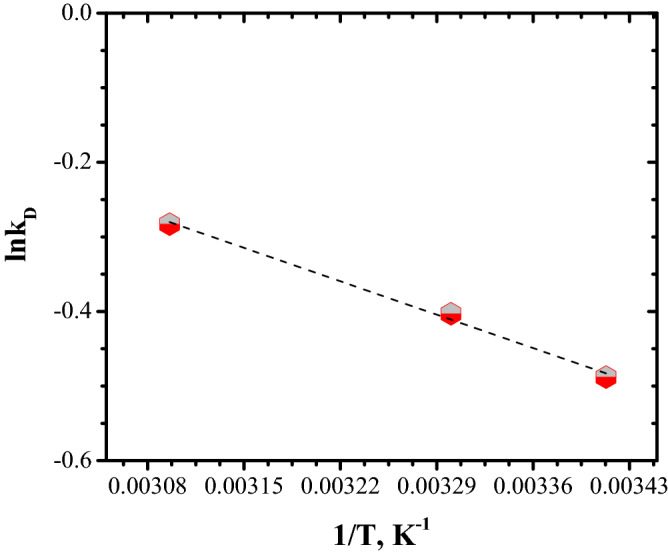
Van't Hoff plot of the adsorption of Congo Red dye on FA.

From Fig. 17, it can be noted that the effect of temperature has a positive impact on adsorption capacity: its value increases as temperature increases from 293 to 323 K. Further, the data obtained by plotting ln k_D_ vs. 1/T were subject for thermodynamic study in order to establish the nature of adsorption process, Fig. 18 and Table 5 shows the thermodynamic parameters of Congo Red dye adsorption on FA.

**Table 5:** Thermodynamics parameters for Congo Red dye adsorption.

∆G° (kJ·mol^−1^)			∆H° (kJ mol^−1^)	∆S°(kJ mol^−1^·K^−1^)
298 K	303 K	323 K		
− 4.15	− 4.3	− 4.57	5.32	14.14

The values obtained for Gibbs free energy (ΔG°) demonstrate that Congo Red dye adsorption onto FA is favorable and spontaneous. The positive value obtained for ∆S° indicates a good attraction of Congo Red dye by FA surface. The negative value for ∆H° signifies that the adsorption process is exothermic.

### Regeneration and stability investigations

**Figure 19:**
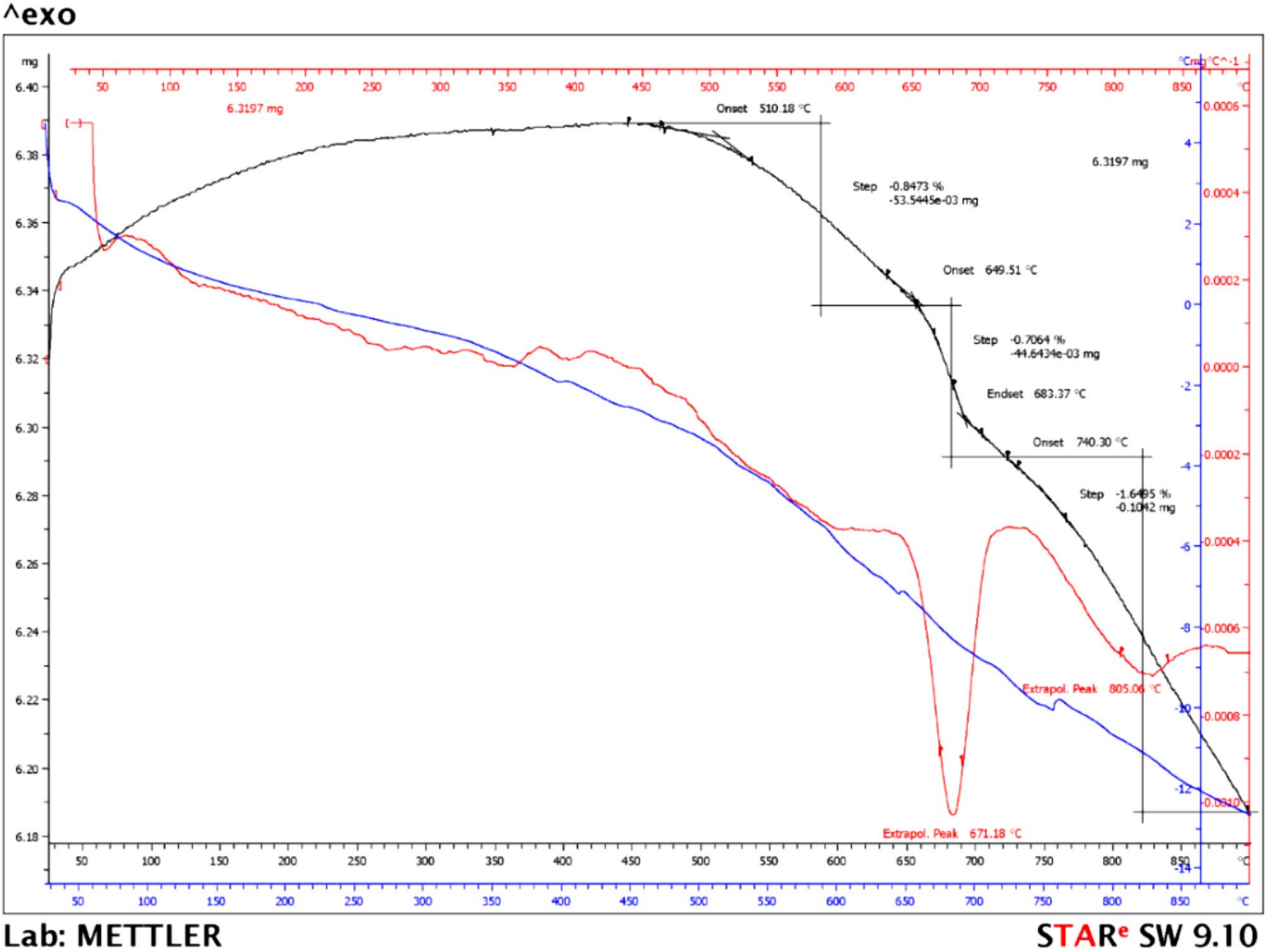
TGA of fly ash after Congo Red dye adsorption.

**Figure 20:**
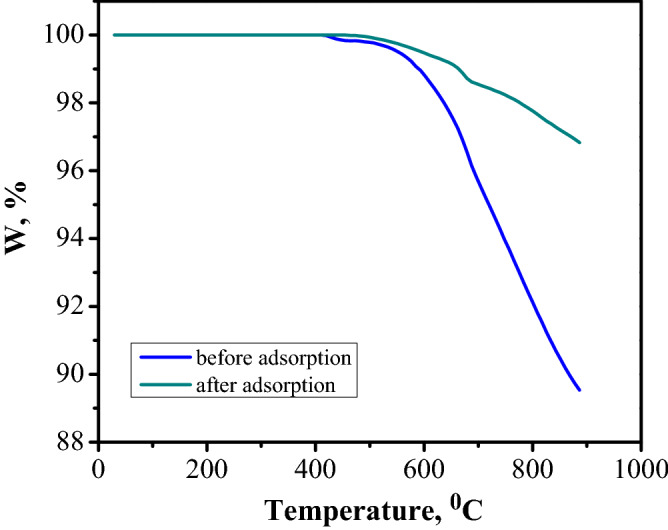
Comparison of TGA curves.

The regeneration capacity of FA adsorbent decides the cost and process efficiency and plays a main role in its large application. Before establishing the regeneration method, a thermal study was realized, TGA analysis was conducted to compare the differences in adsorbent before adsorption (Fig. 7) and after CR dye adsorption. Figure 19 shows the TGA analysis after CR dye adsorption while a comparison between the TGA curves of adsorbent in pristine and after CR dye adsorption is depicted in Fig. 20. The data obtained revealed that the FA after adsorption can be regenerated by calcination at relatively low temperature. The TGA curve of FA before CR dye adsorption showed rapid weight loss at 500 °C. According to the above, it can be concluded that the dye was adsorbed by the functional groups of the FA adsorbent.

In this study, on the base of TG analysis, the regeneration was conducted by calcinations of FA after adsorption at 500° for 2 h. From the beginning it should be noted that regeneration based on alkaline, i.e. sodium hydroxide or acids, i.e. hydrochloric acid, nitric acid have not been used because by applying one of these reagents, significant changes at the FA structure can be obtained.

**Figure 21:**
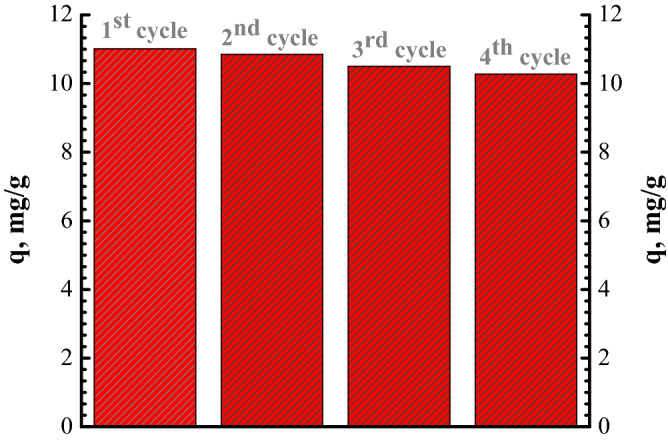
Adsorption capacity in regeneration process.

During calcination, the Congo Red retain onto FA was transformed by oxidative decomposition together with water desorption. Adsorption studies onto regenerated FA were carried out four times to confirm the performance in CR removal. The adsorption capacity after four cycles of regeneration is presented in Fig. 21. After four cycles, the FA exhibited about 10% lower adsorption capacity. This decrease can be ascribed to modification of specific surface area, this decreasing by repeated calcinations. The lower decrease in adsorption capacities can be explain due to a change in surface characteristics and/or pore collapse during the calcination process. After first regeneration, the adsorption capacity persisted constant. The findings of the experiments indicate that FA may be regenerated by calcination with an acceptable adsorption capacity, which leads that FA material in unmodified form proposed in this study shows a good stability. These results are in accord with literature^50^.

**Figure 22:**
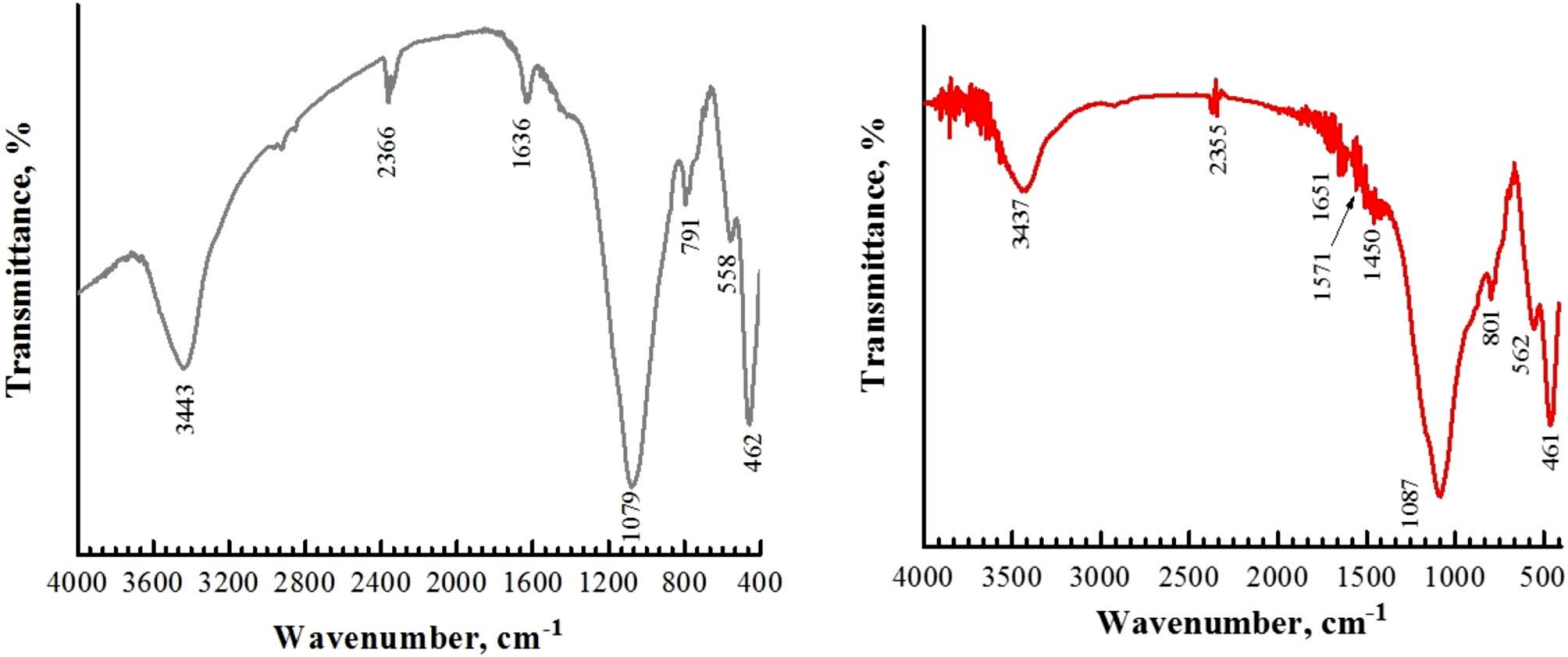
FTIR spectra: (left) before adsorption; (right) after adsorption.

Figure 22 shows the FTIR analysis of FA material before and after Congo Red dye adsorption onto fly ash regenerated 4th cycles.

After adsorption process it can be noted some shifts of the characteristic peaks. For example, the shift of the peak from 3443 to 3437 cm^−1^ is possible generated by the action of OH- bond with CR dye. Also, from the Fig. 22 the shifts of peaks from 2366 cm^−1^, 1636 cm^−1^, 1079 cm^−1^, and 791 cm^−1^ to 2355 cm^−1^, 1651 cm^−1^, 1087 cm^−1^, and 801 cm^−1^ can be attributed to electrostatic interaction between FA surface and CR dye. The shifts and the reduction of their amplitude demonstrates that Congo Red dye reacted with surface of FA.

The quantity of Congo Red dye adsorbed is given on the other hand through a series of new bands. In particular, the peak at ~ 1450 cm^−1^ completely confirms the N=N stretching vibration corresponding to Congo Red dye^51,52^. It can be concluded that all the changes, i.e. the shifts of the peaks and the appearance of new peaks as well clearly evident about the successfully adsorption of Congo Red dye onto the surface of FA.

### Congo Red dye adsorption mechanism

**Figure 23:**
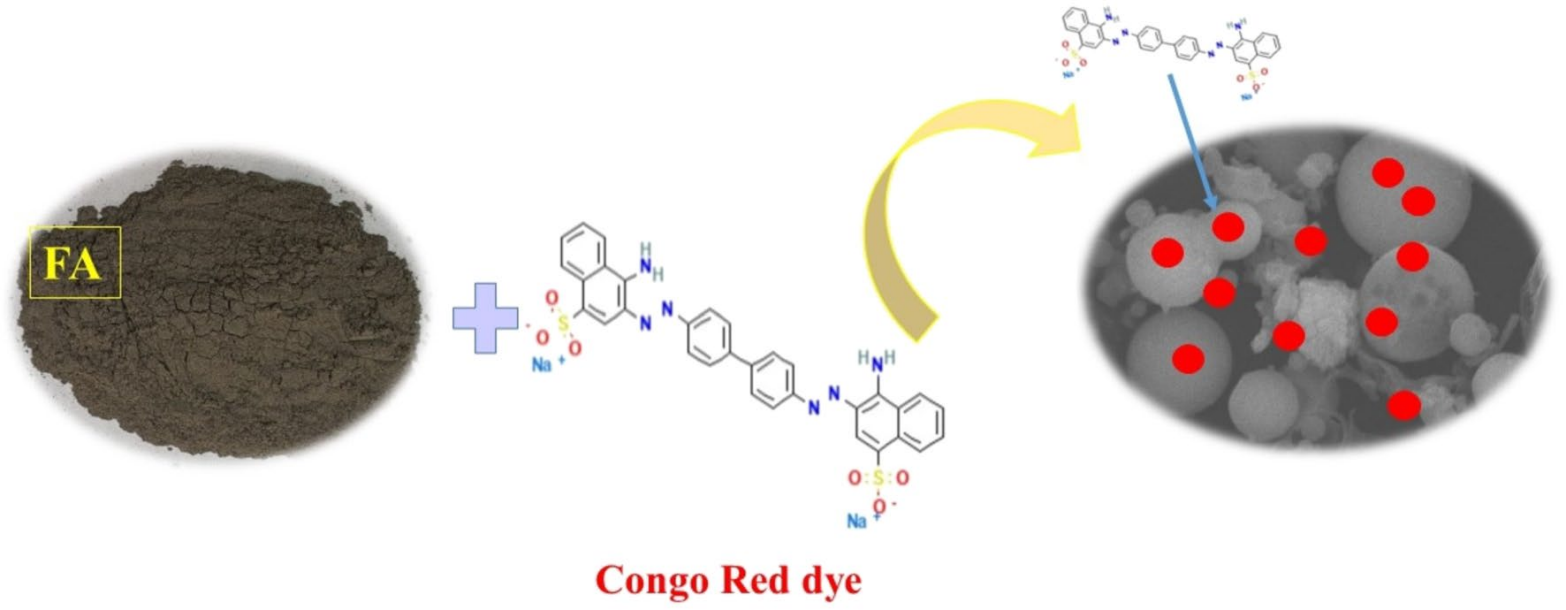
Adsorption mechanism of CR dye adsorption by FA.

Mahmoodi et al. pointed out in their great research that several parameters of adsorbent and adsorbate have an impact on the kinetic and quantity of removal of adsorbate. The adsorption mechanism of Congo Red dye by FA (Fig. 23), can be explain through strong electrostatic attraction between mainly positive surface charge of FA and the negative charge of CR dye^53,54^.

On the other hand, Fig. 16 shows the Langmuir and Freundlich linear plots for CR adsorption onto FA. As can be observed, both models match experimental data well, with R^2^ values around 0.90. The application of both isotherms demonstrates that monolayer homogenous adsorption and heterogeneous energetic distribution of active sites on the adsorbent's surface occur at the same time as adsorption onto the FA. One of the key reasons for heterogeneous adsorption and monolayer process is high-energy adsorption sites. Another factor is the surface condensation of liquid adsorbates. The first two levels interact with the surface, whereas molecules beyond the first two layers interact with one other, resulting in multilayer adsorption. The mechanism of dye adsorption is complicated, and both homogeneous and heterogeneous adsorption occur simultaneously in this adsorption process, according to the aforementioned arguments. The adsorption process can, however, be better described using the Langmuir model.

### Comparison between maximum adsorption capacities of CR dye adsorption of FA and other adsorbents

Table 6 shows a comparison between FA and other adsorbents used for CR dye adsorption.

**Table 6:** Comparison between FA and other adsorbents.

Adsorbent	Adsorption capacity, mg/g	References
Calcium-rich fly ash	9.41	^55^
Bagasse fly ash [BFA]	11.885	^56^
Untreated Bottom Ash	24.36	^57^
Physical activated bottom ash	106.61	^57^
Fly ash/NiFe_2_O_4_ composites [FANiFe_50_]	22.73	^50^
Bricks Kiln Chamber Fly Ash	33.3	^58^
Synthesised zeolites from fly ash [ZS1, ZS2, ZS3, ZS4, ZS5, ZS6, ZS7, CZX]	110.24; 132.52; 140.26, 128.56; 132.52; 146.53; 162.35; 88.64	^59^
Zeolite/algae composite [ZPG]	12.25	^60^
Padina gymnospora [PG]	12.38	^60^
Zeolite [Z]	9.23	^60^
FA	22.12	Present study

The adsorption capacity depended on the working conditions applied in the adsorption process (pH, initial concentration, adsorbent dose, etc.) and by the properties of the adsorbent.

## Materials and methods

### Materials

The Holboca thermo electrical power plant (Iasi, Romania) provided the FA. All the reagents involved in the adsorption study were acquired from Sigma-Aldrich and were used as received.

**Figure 24:**
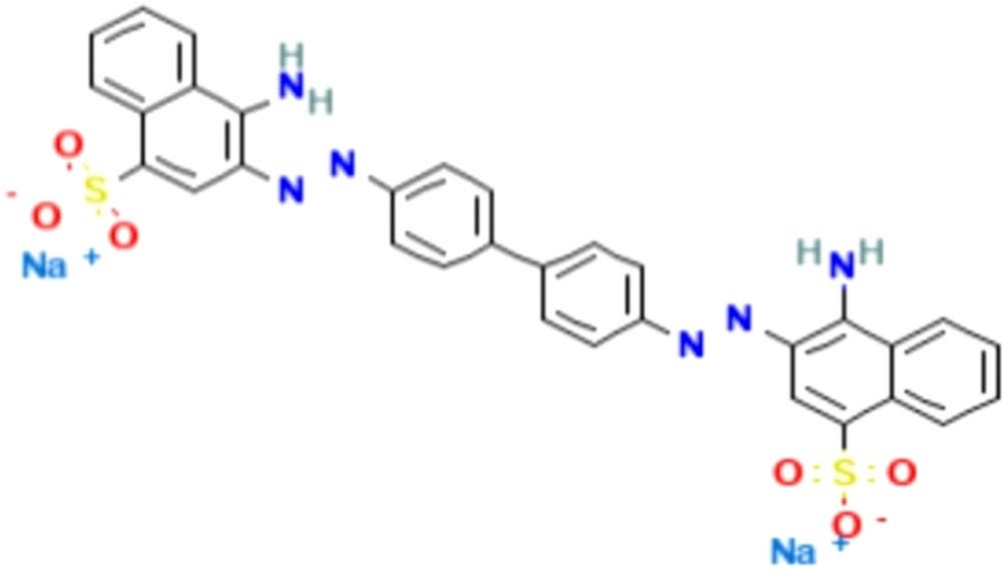
Structure of Congo Red dye.

The structure of Congo Red is presented in Fig. 24^39^.

### FA characterisation

The properties of the materials were established through Scanning electron microscopy (SEM), Energy dispersive X-ray spectroscopy (EDX), X-ray diffraction (XRD), Fourier transform spectroscopy (FTIR), Brunauer–Emmett–Teller (BET) surface area analysis and thermogravimetric analysis (TGA):

SEM by Vega Tescan LMH II;

EDX analyses were determined using a Bruker EDAX XFlash detector;

A diffractometer type X’PERT PRO MRD was used for XRD analysis;

FTIR analysis was realised with a Thermo Scientific Nicolet 6700 spectrometer;

BET analysis was by Quantachrome Autosorb 1-MP;

TGA analysis was investigated with Mettler Toledo TGA/SDTA 851;

A Hanna pH-meter was used for pH measurement.

### Adsorption studies

The influence of three parameters was analysed using batch adsorption experiments (intermittent stirring). All tests were realised at room temperature and natural pH. The initial solutions, of 10–50 mg/L were obtained by diluting the Congo Red stock solution of 1 g/L with deionised water. A DR3900 laboratory spectrophotometer (Hach) was used for CR dye analysis at the absorbance of 498 nm.

## Conclusion

The main conclusions to be drawn:

- The current research showed the possibility of applying unmodified fly ash as adsorbent for Congo Red dye.
- Different characterisation methods were applied: SEM, EDX, XRD, FTIR and BET surface area analysis and TGA.
- The influences of adsorbent dose, contact time, temperature and initial CR dye concentration were studied. The results indicated that the CR dye adsorption capacity improved with increasing CR initial concentration, temperature and contact time. Otherwise, the adsorption capacity diminished with increasing adsorbent dose.
- The kinetics of the CR dye adsorption processes showed rapid adsorption, a contact time of 60 min being enough to reach equilibrium.
- The best fit with experimental data was obtained by applying the PSO kinetics model.
- The Langmuir model fit better the adsorption results compared to the Freundlich model, with ah adsorption capacity of 22.12 mg/g.
- Thermodynamic study revealed that the process is favourable, spontaneous, and exothermic.
- The regeneration investigation indicates that FA material in unmodified form proposed in this study shows a good stability.
